# Supplementary material for: DMRforPairs: identifying Differentially Methylated Regions between unique samples using array based methylation profiles
Source: BMC Bioinformatics. 2014 May 15;15:141. doi: 10.1186/1471-2105-15-141 (PMC4046028; doi:10.1186/1471-2105-15-141)
Supplement: Additional file 2 — DMRforPairs output for the comparison of A431-MCF7 and NA17018-NA17105. Please start from the HTML files in each folder. Available via the BMC Bioinformatics website. [file 1471-2105-15-141-S2.zip › 1394847754114233_MOESM2_ESM/A431_MCF7/figures/30880.pdf]

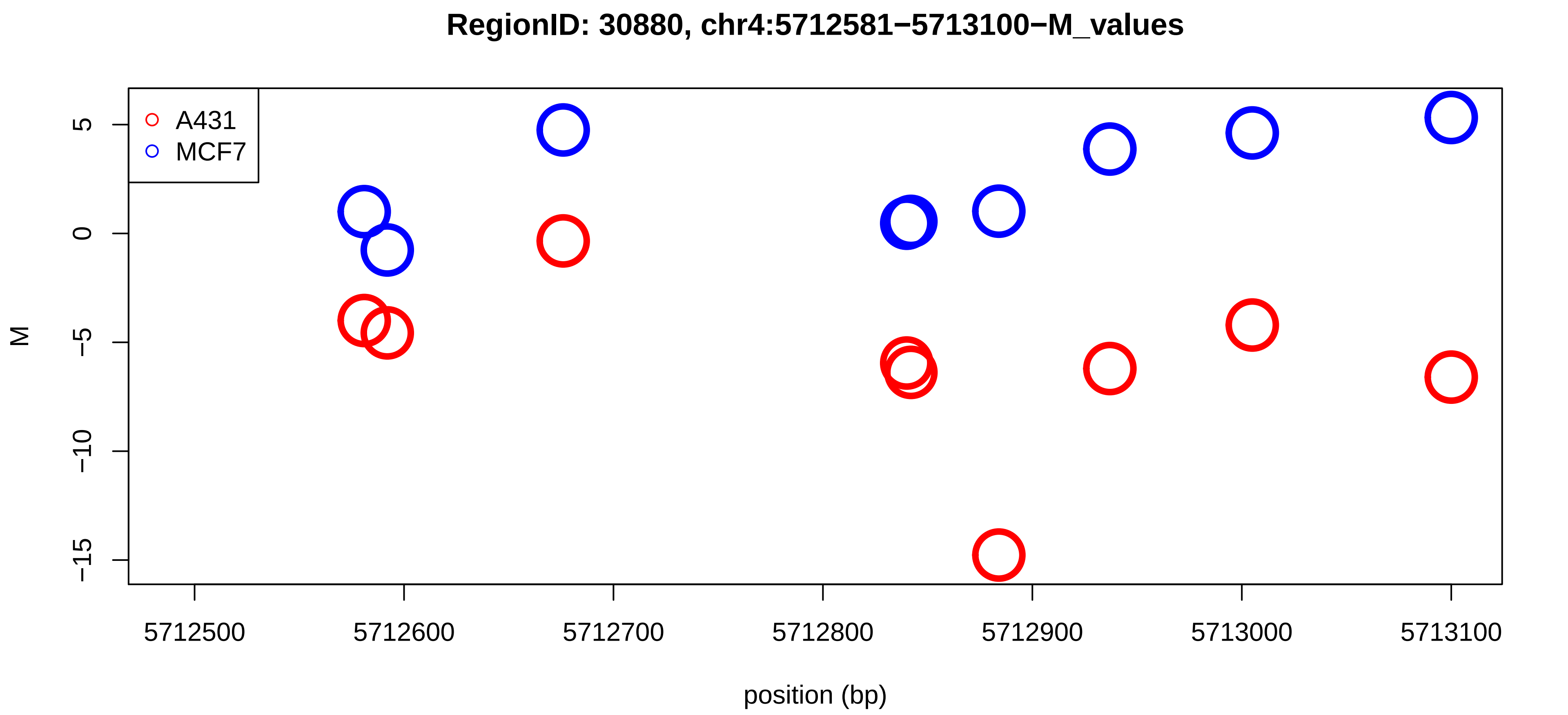
RegionID: 30880, chr4:5712581−5713100−M_values
A431
MCF7
M
5
0
−5
−10
−15
5712500
5712600
5712700
5712800
5712900
5713000
5713100
position (bp)

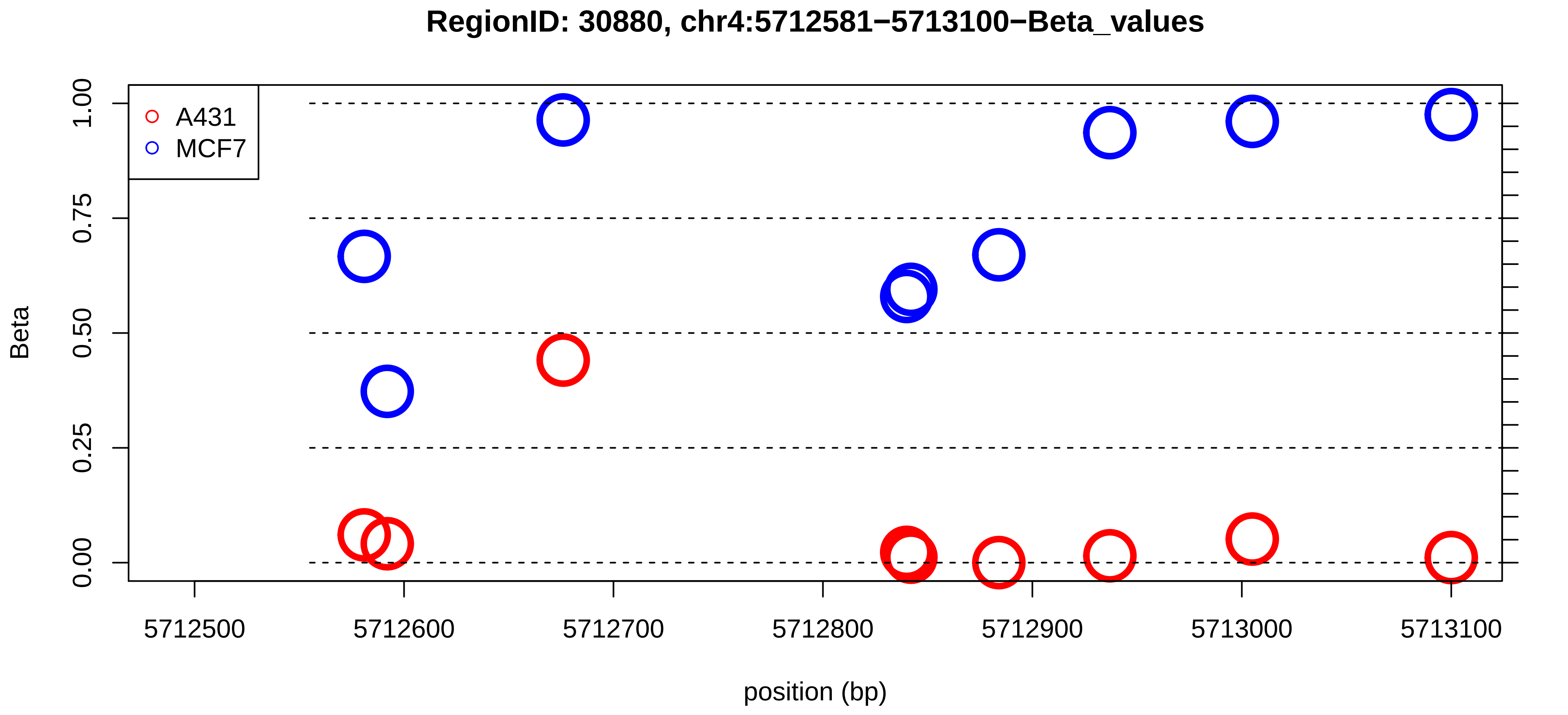
RegionID: 30880, chr4:5712581−5713100−Beta_values
A431
MCF7
Beta
1.00
0.75
0.50
0.25
0.00
5712500
5712600
5712700
5712800
5712900
5713000
5713100
position (bp)

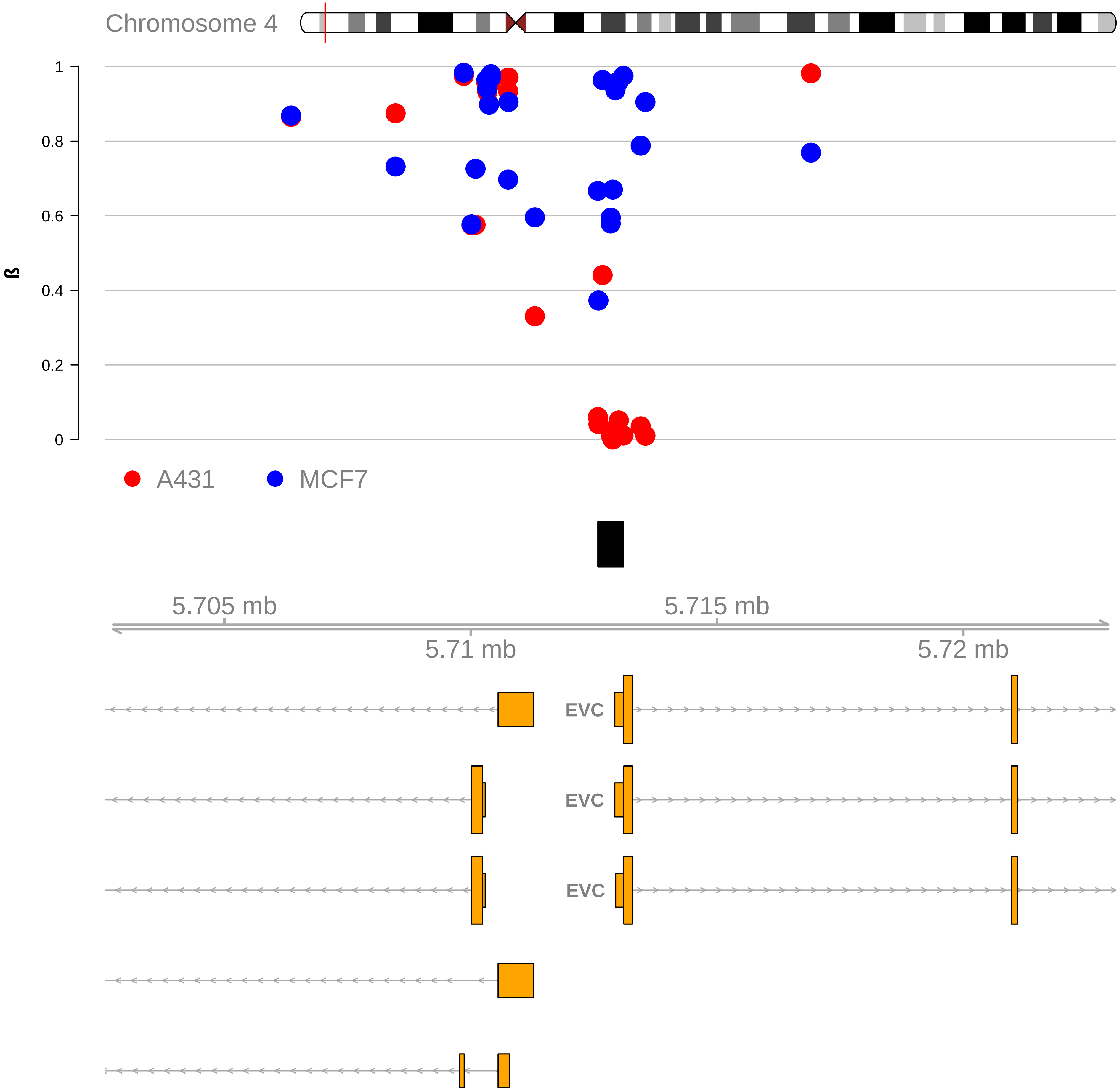

Chromosome 4
1
0.8
0.6
0.4
0.2
0
ß
A431
MCF7
5.705 mb
5.715 mb
5.71 mb
5.72 mb
EVC
EVC
EVC
